# Supplementary material for: WordCommentsAnalyzer: A windows software tool for qualitative research
Source: F1000Res. 2018 Sep 4;7:536. Originally published 2018 May 3. [Version 2] doi: 10.12688/f1000research.14819.2 (PMC6137411; doi:10.12688/f1000research.14819.2)
Supplement: Supplementary file 3 [file f1000research-7-17614-s0002.tgz › b2a79496-4ada-47c4-9682-a99ce867836d.zip!/Eight Iranian Health Journals/Archives of Rehabilitation_25.docx]

© Archives of Rehabilitation. This work is licensed under CC BY-NC 4.0 (https://creativecommons.org/licenses/by-nc/4.0/).

Volume & Issue: دوره 13 - شماره 4

Title: بررسی تکرارپذیری ثبات وضعیتی دینامیک در افراد سالم و بیماران کمردرد غیر اختصاصی

http://rehabilitationj.uswr.ac.ir/article-1-727-fa.html

هدف: تحقیق حاضر به بررسی تکرارپذیری ثبات پوسچرال در شرایط دینامیک کلی بدن می‌پردازد تا بتوان در ارزیابی و درمان ضایعات از آن استفاده کرد. روش‌ بررسی: در این اعتبارسنجی، ۱۲ فرد سالم و ۱۲ بیمار کمردردی به صورت نمونه‌گیری غیراحتمالی ساده ۸ وضعیت آزمون خم و راست شدن تنه را در شرایط متفاوتی از سرعت حرکت، راستای حرکت و وجود یا عدم وجود بار خارجی انجام دادند. در فاصله زمانی ۷ تا ۱۰ روز تکرار آزمون‌ها صورت گرفت. داده‌های صفحه نیرو جهت استخراج پارامترهای انحراف معیار مقدار جابجایی مرکز فشار و سرعت جابجایی در دو جهت قدامی‌خلفی و طرفی، میانگین کلی سرعت و طول مسیر مورد محاسبه قرار گرفت. تکرارپذیری نسبی با استفاده از ضریب همبستگی درون گروهی و تکرارپذیری مطلق با استفاده از خطای معیار اندازه‌گیری و ضریب تغییرات مورد ارزیابی قرار گرفت. یافته‌ها: از میان پارامترهای مختلف مرکز فشار میانگین کلی سرعت در همه آزمون‌ها تکرارپذیری متوسط تا بالایی را نشان داد (دامنه آی.‌سی.‌سی. ۰/۹۱-۰/۶). در افراد سالم پارامترهای دیگری مثل انحراف معیار مقدار جابجایی مرکز فشار و انحراف معیار سرعت در جهت قدامی‌خلفی، نیز تکرارپذیری خوبی را نشان دادند (دامنه آی.‌سی.‌سی. ۰/۹۷-۰/۶۶). در افراد کمردردی در شرایط انجام آزمون‌های قرینه انحراف معیار مقدار جابجایی مرکز فشار در جهت قدامی‌خلفی ( دامنه آی.‌سی.‌سی. ۰٫۷۵-۰٫۶۱)، و در شرایط غیر قرینه انحراف معیار مقدار جابجایی مرکز فشار در جهت طرفی، تکرارپذیری خوبی را از خود نشان دادند. (دامنه آی.‌سی.‌سی. ۰/۸۱-۰/۶۲). نتیجه‌گیری: با توجه به تکرارپذیری بالا، به نظر می‌رسد میانگین کلی سرعت مرکز فشار در شرایط دینامیک، پارامتر خوبی برای ارزیابی فعالیت‌های دینامیک مثل باربرداری و حرکات ترکیبی در شرایط ارزیابی بالینی در بیماران و افراد سالم باشد.

Title: بررسی آگاهی واجشناختی در کودکان مبتلا به اختلالات واجی

http://rehabilitationj.uswr.ac.ir/article-1-1161-fa.html

هدف: ارتباط بین آگاهی واج‌شناختی و اختلالات واجی در دهه‌های اخیر مورد توجه قرار گرفته است. نقص در توانایی‌های واجی‌ می‌تواند دلیل اختلالات آگاهی واج‌شناختی در کودکان مبتلا به اختلال واجی باشد. در مطالعه حاضر تلاش شد که نقایص آگاهی واج‌شناختی در کودکان مبتلا به اختلالات واجی مورد بررسی قرار گیرد. روش بررسی: پژوهش از نوع مقطعی و مقایسه‌ای بود. نمونه پژوهش شامل ۲۵ کودک مبتلا به اختلالات واجی و ۲۵ کودک طبیعی بود که کودکان دارای اختلال به شیوه در دسترس و کودکان طبیعی از طریق همسان‌سازی با گروه اختلال انتخاب شدند. ابزارهای پژوهش برای تعیین اختلالات واجی آزمون اطلاعات آوایی و برای بررسی آگاهی واج‌شناختی آزمون آگاهی واج‌شناختی بود. آگاهی از ترکیب و تقطیع هجا و قافیه و تجانس مورد بررسی قرار گرفت. داده‌های حاصل با استفاده از آزمون‌های کای‌اسکوئر، تی مستقل و تی جفت تجزیه و تحلیل شد. یافته‌ها:‌ میانگین آگاهی واجی در کودکان با اختلالات واجی به صورت معنی‌داری پایین‌تر از کودکان طبیعی بود (۰/۰۰۱>P). تفاوتی‌ میان دو گروه از نظر آگاهی از تقطیع (۰/۰۷۳=P) و ترکیب هجا (۰/۱۸۹=P) وجود نداشت، اما از نظر آگاهی از قافیه (۰/۰۰۱>P) و تجانس (۰/۰۰۱>P) تفاوت بین دو گروه معنی‌دار بود. نتیجه‌گیری: یافته‌های حاصل از پژوهش نشان داد که کودکان مبتلا به اختلالات واجی از نظر آگاهی واج‌شناختی در سطح پایین‌تری از کودکان طبیعی قرار دارند. با توجه به نتایج حاصل از این پژوهش ‌می‌توان به آسیب‌شناسان گفتار و زبان توصیه نمود که هنگام درمان کودکان مبتلا به اختلالات واجی، آگاهی واج‌شناختی را هم مد نظر قرار دهند.

Title: میزان آگاهی مددجویان و درمانگران از مفاد

http://rehabilitationj.uswr.ac.ir/article-1-1073-fa.html

هدف: علیرغم وجود مطالعات قابل توجه در مورد آگاهی و رعایت منشور حقوق بیماران در کشور، مطالعات اندکی در این خصوص در حوزه توانبخشی اجرا و گزارش شده است. هدف از این پژوهش بررسی آگاهی مددجویان و درمانگران توانبخشی از منشور حقوق بیماران در فاز درمان است. روش بررسی: این مطالعه توصیفی-تحلیلی به روش مقطعی در دو مرکز وابسته به دانشگاه علوم بهزیستی و توانبخشی در سال ۹۰-۱۳۸۹ انجام شد. نمونه‌گیری به روش در دسترس شامل ۱۰۳مراجعه کننده و ۲۸ درمانگر بود. ابزار جمع‌آوری داده‌ها پرسش‌نامه در دو فرم درمانگران و مراجعین بود که روایی و پایایی آن تایید شد. داده‌ها با استفاده از ضریب همبستگی پیرسون مورد تجزیه و تحلیل قرار گرفتند. یافته‌ها: ۴۴ زن و ۵۹ مرد مراجع و ۲۰ زن و ۸ مرد درمانگر در این مطالعه حضور داشتند. سطح آگاهی ٪۴۷/۶ مراجعین از مفاد منشور حقوق بیماران ضعیف، ٪۱۶/۵ متوسط و ٪۳۵/۹ خوب بود. ارتباط معنی‌داری بین سن، جنس، محل مراجعه و سطح آگاهی مراجعین پیدا نشد. اما ارتباط معنی‌داری بین سطح آگاهی آنان و سطح تحصیلات، تعداد دفعات مراجعه و مراجعه به واحد توانبخشی وجود داشت (۰/۰۵>P). سطح آگاهی ٪۵۳/۶ درمانگران از مفاد منشور حقوق بیماران خوب، ٪۱۴/۳ متوسط و ٪۳۲/۱ ضعیف بود. ارتباط معنی‌داری بین سن، جنس، مقطع تحصیلی، رشته تحصیلی و سنوات با سطح آگاهی وجود نداشت، ولی ارتباط معنی‌داری بین سطح آگاهی و محل خدمت درمانگران مشاهده شد (۰/۰۱>P). نتیجه‌گیری: با توجه به این که مددجویان توانبخشی آسیب‌پذیر‌‌ترین گروه بیماران هستند و از شرایط ویژه‌تری نسبت به مراجعین به سایر بخش‌‌‌های خدمات درمانی برخوردارند، ارتقاء سطح کارایی توانبخشی و رضایت‌مندی مراجعین آن، مستلزم ارتقاء سطح آگاهی عمومی ‌نسبت به خدمات توانبخشی و حقوق مددجویان و تحقیق و طراحی راهکار‌‌های رعایت حقوق مددجویان توسط سیاست‌گزاران نظام خدمات سلامت و خصوصاً توانبخشی است .

Title: مدل‌سازی مکانیکی حرکت اکستنشن و فلکشن مفصل آرنج در زمینه توانبخشی

http://rehabilitationj.uswr.ac.ir/article-1-1008-fa.html

هدف: هدف این تحقیق بهینه کردن حرکت توانبخشی اکستنشن و فلکشن مفصل آرنج، از لحاظ اتلاف انرژی و صدمات ناشی از تنش‌‌های وارده از طرف تجهیزات حرکتی به مفاصل و اندام‌‌های درگیر در حرکت، با استفاده از بررسی تغییرات خواص ویسکوالاستیک در انجام حرکت موردنظر،به ازای تغییر سرعت انجام حرکت بود. روش بررسی: در این مطالعه کاربردی جمعیت آماری مختصر به ۵ نفرمرد سالم بدون هیچگونه سابقه و مشکل در سیستم عصبی-عضلانی و با لحاظ کردن شاخص توده‌بدنی بود. بر روی این افراد تست (CPM) گشتاور پسیو توسط دستگاه ایزوکینتیک سایبکس (CYBEX) در حرکت اکستنشن و فلکشن مفصل آرنج در ۴ سرعت متفاوت (۷۵ ،۴۵ ، ۱۵، ۱۲) در ۵ سیکل متوالی در دامنه حرکتی ۰ تا ۱۳۰ درجه، بر روی دست چپ هر فرد انجام شد و سپس اطلاعات بدست آمده از تست‌‌های انجام شده برای تحلیل و آنالیز، وارد نرم‌افزار ریاضی و تحلیلی (MATLAB) شد. به منظور مدل‌سازی حرکت و بررسی اثر خواص ویسکوالاستیک در حرکت موردنظر و هم‌چنین محاسبه پارامتر‌‌های بیومکانیکی از منحنی‌‌های گشتاور پسیو بدست آمده از آزمایش‌ها، از یک مدل مکانیکی ویسکوالاستیک پسیو سه اِلمانی استفاده شد و برای استخراج ضرایب ویسکوز و الاستیک مدل، روابط ریاضی مدل مربوطه را با منحنی‌‌های گشتاور پسیو فیت کردیم. یافته‌‌ها: افزایش میزان سرعت حرکت باعث افزایش میزان پارامتر‌‌های بیومکانیکی کار، هیسترسیس و ضرایب الاستیک، کاهش ضریب ویسکوز، افزایش میزان اتلاف انرژی و افزایش مقاومت المان‌‌های ویسکوز شد. هم‌چنین پاسخ مدل مکانیکی، بیانگر سازگاری عملکرد مدل موردنظر با عملکرد مکانیکی اندام‌‌های درگیر در طول حرکت بود. نتیجه‌گیری: نتیجه این تحقیق حاکی از انجام حرکات توانبخشی در سرعت‌‌های پایین و استفاده از تجهیزات توانبخشی با نیروی مقاوم ویسکوالاستیک به منظور بهینه کردن حرکت و کاهش صدمات وارده در بافت‌ها و مفاصل درگیر بود.

Title: اثربخشی آموزش مهارت زندگی بر سلامت روان دانش‌آموزان کم شنوا

http://rehabilitationj.uswr.ac.ir/article-1-1226-fa.html

هدف: هدف این پژوهش بررسی اثربخشی آموزش مهارت زندگی بر سلامت روان دانش‌آموزان کم‌شنوای شهرستان‌های تهران بود. روش بررسی: پژوهش حاضر، یک مطالعه آزمایشی با طرح پیش‌آزمون-پس‌آزمون و گروه کنترل بود. جامعه آماری این پژوهش را دانش‌آموزان کم‌شنوای دوم و سوم متوسطه شهرستان‌های تهران تشکیل دادند. برای انتخاب آزمودنی‌ها از روش نمونه‌گیری در دسترس استفاده شد، به طوری که در این پژوهش ۴۰ دانش‌آموز شرکت داشتند. آزمودنی‌ها به صورت تصادفی به دو گروه ۲۰ نفری تقسیم شدند (یک گروه آزمایش و یک گروه کنترل). گروه آزمایشی، آموزش مهارت‌های زندگی را در ۹ جلسه دریافت کردند در حالی که به گروه کنترل این آموزش ارائه نشد. ابزارهای استفاده شده در این پژوهش آزمون هوشی وکسلر بزرگسالان و پرسش‌نامه سلامت عمومی بود. اطلاعات جمع‌آوری شده با استفاده از تحلیل کوواریانس چندمتغیری تحلیل شد. یافته‌ها: نتایج نشان داد که بعد از مداخله، میانگین نمرات سلامت روان گروه آزمایش به طور معناداری بیشتر از گروه کنترل بود (۰/۰۵>P). هم‌چنین نمرات سلامت روان گروه آزمایش در خرده‌مقیاس‌های علایم جسمانی، اضطراب، نقص در عملکرد اجتماعی و افسردگی معنادار بود (۰/۰۵>P). نتیجه‌گیری: برنامه آموزشی مهارت زندگی باعث بهبود سلامت روان دانش‌آموزان کم‌شنوا شد و علایم جسمانی، اضطراب، نقص در عملکرد اجتماعی و افسردگی آنان را کاهش داد. بنابراین برنامه‌ریزی برای ارائه آموزش مهارت زندگی به این دانش‌آموزان اهمیت ویژه‌ای دارد.

Title: بررسی علل ژنتیکی کم توانی ذهنی در استان بوشهر

http://rehabilitationj.uswr.ac.ir/article-1-999-fa.html

هدف: عقب‌ماندگی ذهنی شدید تقریباً در ٪۵۰ موارد دارای علتی ژنتیکی می‌باشد. در این مطالعه به منظور درک بهتر این عوامل و هم‌چنین کمک به انجام مشاوره ژنتیکی موثرتر، عوامل ژنتیکی این بیماری در استان بوشهر بررسی شد. روش بررسی: در این پژوهش توصیفی که از نوع مطالعات مقطعی-کاربردی است، با کمک سازمان بهزیستی استان بوشهر از بین ۲۰۰ خانواده‌ دارای دو فرزند مبتلا به عقب‌ماندگی ذهنی و ‌یا بیشتر که بیماری آنها توسط پزشک تأیید شده بود، ۶۹ خانواده با کمک روش نمونه‌گیری ساده انتخاب شدند. سپس از تمام افراد سالم و مبتلا در خانواده‌های انتخاب شده خونگیری صورت گرفت. یافته‌ها: در هیچیک از موارد بررسی شده ناهنجاری‌های کروموزومی‌ مشاهده نشد. یک خانواده دچار نشانگان ایکس شکننده بود. پس از بررسی‌های آنالیز پیوستگی در ۱۸ خانواده‌ دارای فرزندان میکروسفال از نوع اتوزومی ‌مغلوب اولیه، ۶ خانواده به جایگاه‌های مربوطه (ام.سی.پی.اچ) پیوستگی نشان دادند. ‌یک خانواده دچار میکروسفالی از نوع سندرمی‌ بود. دو خانواده‌ میکروسفال و‌ یک خانواده‌ دچار عقب‌ماندگی ذهنی از نوع غیر‌سندرمی‌ و غیر‌میکروسفال، طرح توارث اتوزوم غالب را نشان دادند. نتیجه‌گیری: براساس نتایج بدست آمده در این ‌بررسی ناتوانی ذهنی بسیار هتروژن می‌باشد و عقب‌ماندگی ذهنی با توارث اتوزومی‌ مغلوب همراه با کاهش در اندازه دور سر(میکروسفالی) درصد بسیار زیادی (٪۲۶/۰۹) از موارد عقب‌ماندگی ذهنی ژنتیکی در استان بوشهر را به خود اختصاص داده است.

Title: تحلیل زمان سپری‌شده تا عودهای مکرر در بیماران اسکیزوفرنیک با استفاده از مدل‌های پیشامدهای بازگشتی

http://rehabilitationj.uswr.ac.ir/article-1-898-fa.html

هدف: اسکیزوفرنیا به جهت شیوع بالا و شدت نشانه‌ها و عودهای مکرر مورد تأکید قرار می‌گیرد. در این مطالعه به تحلیل خطر بازگشت عودهای مکرر در بیماران مبتلا به اسکیزوفرنیا به وسیله مدل‌های پیشامدهای بازگشتی پرداخته شده است. روش بررسی: نوع مطالعه ‌این تحقیق مشاهده‌ای طولی آینده‌نگر بوده است که در آن از اطلاعات ۱۵۹ بیمار مبتلا به اسکیزوفرنیا بستری در مرکز آموزشی-درمانی روانپزشکی رازی استفاده شده است. مدل شرطی نوع دوم و مدل شکنندگی برای تحلیل زمان‌ها تا عود به کار گرفته شده است. نرم‌افزارهای مورد استفاده برای تجزیه و تحلیل داده‌ها SAS نسخه ۹/۱ و R نسخه ۲/۱۱/۰ بود. سطح معنی‌داری آزمون‌ها نیز ۰/۰۵ در نظر گرفته شده است. یافته‌ها: در مدل شرطی نوع دوم متغیرهای سن در آغاز بیماری (۰/۰۲۱=P) و وضعیت تأهل (۰/۰۰۱>P) تنها بر خطر وقوع عود اول بیماری دارای اثر معنی‌دار بودند و هیچ یک از عوامل سن در آغاز بیماری، جنسیت، وضعیت تأهل، نحوه شروع بیماری، سابقه ضربه به سر و سابقه بیماری اسکیزوفرنیا در خانواده بر خطر وقوع عود دوم اثر معنی‌دار نداشتند. سابقه ضربه به سر نیز تنها بر خطر وقوع عود سوم بیماری اثر معنی‌دار داشت (۰/۰۲۰=P). در مدل شکنندگی، تنها اثر متغیرهای جنس (۰٫۰۱۴=P) و سابقه ضربه به سر (۰٫۰۱۴=P) بر خطر وقوع عودهای بعدی معنی‌دار شد. نتیجه‌گیری: برخی عوامل تحت مدل شرطی نوع دوم دارای اثر معنی‌دار بر خطر وقوع عودهای بعدی در بیماران مبتلا به اسکیزوفرنیا بودند و اثر برخی دیگر تحت مدل شکنندگی، معنی‌دار شد. نیاز است در پژوهش‌های بعدی، اثر دیگر متغیرهای پیش‌آگهی‌دهنده بر خطر وقوع یا زمان وقوع عودهای بعدی مورد بررسی قرار گیرد.

Title: اثربخشی مداخله شناختی رفتاری گروهی بر اصلاح تصویر تن و افزایش عزت نفس در زنان مبتلا به سرطان پستان پس از جراحی ماستکتومی

http://rehabilitationj.uswr.ac.ir/article-1-925-fa.html

هدف: پژوهش حاضر با هدف اصلاح تصویر تن و افزایش عزت نفس بیماران مبتلا به سرطان پستان پس از جراحی ماستکتومی از طریق مداخله شناختی رفتاری گروهی انجام گرفته است. روش بررسی: جامعه آماری پژوهش، بیماران مبتلا به سرطان پستان ۳۰ تا ۶۰ ساله مراجعه کننده به بیمارستان امام حسین (ع) تهران بود که پس ازجراحی ماستکتومی ‌تحت پرتو درمانی قرار داشته‌اند. اعضای گروه نمونه از طریق نمونه‌گیری مبتنی بر هدف انتخاب و با استفاده از گمارش تصادفی به دو گروه آزمایش (۱۰) و کنترل (۱۳) تقسیم شدند. اعضای هر دو گروه در پیش‌آزمون و پس‌آزمون براساس مقیاس تصویر تن و روابط هورمز و همکاران و عزت نفس آلیس پوپ مورد ارزیابی قرار گرفتند. گروه آزمایش در معرض «مداخله شناختی رفتاری گروهی» در قالب دوازده جلسه ۹۰ دقیقه‌ای (دو جلسه در هفته) قرار گرفت. پس از جمع‌آوری داده‌ها در پس‌آزمون، داده‌ها با استفاده از تحلیل کواریانس با هدف حذف اثر پیش‌آزمون بر پس‌آزمون و تحلیل واریانس برای بررسی تفاوت گروه‌ها پردازش شد. یافته‌ها: میانگین تصویر تن و عزت نفس در بیماران مبتلا به سرطان پستان ماستکتومی ‌شده پس از اجرای مداخله، در گروه آزمایش بیشتر از میانگین گروه کنترل بوده و تفاوت‌ها از لحاظ آماری معنادار است (۰/۰۱>P). نتیجه‌گیری: به این ترتیب می‌توان نتیجه گرفت که شیوه مداخله مورد استفاده بر اصلاح تصویر تن و افزایش عزت نفس زنان ماستکتومی شده موثر بوده است.

Title: بررسی روایی و پایایی نسخه فارسی پرسشنامه مقیاس سنجش شدت خستگی در افراد مبتلا به مولتیپل اسکلروزیس

http://rehabilitationj.uswr.ac.ir/article-1-1046-fa.html

هدف: هدف از پژوهش حاضر بررسی روایی نسخه فارسی پرسش‌نامه «مقیاس سنجش شدت خستگی» (FSS) در افراد مبتلا به مولتیپل اسکلروزیس می‌باشد. روش بررسی: در تحقیق غیرتجربی و متدولوژیک حاضر ۵۰ نفر (۳۸ زن و ۱۲ مرد)‌ فارسی زبان مبتلا به مولتیپل اسکلروزیس و ۳۰ فرد سالم (۱۸ زن و ۱۲ مرد)‌ با نمونه‌گیری ساده و غیراحتمالی از مراجعین کلینیک سینا از سال ۱۳۸۰ تا ۱۳۸۶ انتخاب و پرسشنامه‌های مقیاس شدت خستگی و ‌۳۶–SF را پاسخ دادند. بررسی تکرارپذیری، در دو نوبت با فاصله حداکثر ۳ هفته انجام شد. همخوانی درونی آیتم‌ها، روایی‌ صوری، همگرا و افتراقی پرسش‌نامه FSS مورد بررسی قرار گرفت. یافته‌ها: هم‌خوانی درونی آیتم‌های پرسش‌نامه « مقیاس سنجش شدت خستگی» با محاسبه ضریب آلفای کرونباخ برابر ۰٫۹۶ می‌باشد که نشان می‌دهد که آیتم‌های مقیاس فوق یک مفهوم را ارزیابی می‌کنند. همبستگی هر آیتم با آیتم‌های دیگر، بعد از اصلاح همپوشانی، بالاتر از حد مطلوب ۰٫۴ بود. ضریب ICC برای بررسی تکرارپذیری نسبی نسخه فارسی مقیاس سنجش شدت خستگی در دفعات آزمون ۰٫۹۳ و بالاتر از مقدار قابل قبول ۰٫۷ بود. پرسش‌نامه FSS و مقیاس قیاس چشمی ‌خستگی با زیرمقیاس سرزندگی ۳۶–SF همبستگی بالایی داشتند (۰/۰۰۰۱=P و ۰/۷۳-=r و ۰/۶۹-=r). ضریب همبستگی با سایر زیر مقیاس‌های ۳۶–SF متوسط بود (۰/۰۰۰۱=P و ۰/۶۵-=r تا ۰/۴۳-=r). جهت بررسی روایی افتراقی، ‌شدت خستگی افراد مبتلا به مولتیپل اسکلروزیس با خستگی معمول افراد سالم فارسی زبان مقایسه شد. نمره‌کلی نسخه فارسی معادل‌سازی ‌شده پرسش‌نامه FSS در افراد سالم (۳/۲۳=M) نسبت به افراد مبتلا به مولتیپل اسکلروزیس (۵/۰۳=M) کمتر می‌باشد نتیجه‌گیری: این یافته‌ها نشان می‌دهد که پرسش‌نامه FSS از پایایی و روایی خوبی برخوردار است و می‌توان با استفاده از آن میزان شدت خستگی افراد مبتلا به مولتیپل اسکلروزیس را بررسی نمود.

Title: تأثیر درمان‌های جراحی بازسازی و بریس عملکردی بر آستانه حس تشخیص حرکت در بیماران مبتلا به پارگی لیگامان متقاطع قدامی حاد

http://rehabilitationj.uswr.ac.ir/article-1-1084-fa.html

هدف: یکی از متداول‌ترین آسیب‌‌های زانو، پارگی لیگامان متقاطع قدامی می‌باشد. حس تشخیص حرکت، بعد از پارگی لیگامان متقاطع قدامی ‌دچار اختلال می‌شود. در این مطالعه سعی داریم با استفاده از روش ترکیبی معادلات برآوردی تعمیم‌یافته، که روشی کارا در تحلیل داده‌‌‌های همبسته می‌باشد به مطالعه تأثیر جراحی بازسازی و بریس عملکردی همراه با کنترل عوامل مخدوشگر در بهبود آستانه حس تشخیص حرکت بیماران با پارگی لیگامان متقاطع قدامی بپردازیم. روش بررسی: این مطالعه شبه‌تجربی با روش نمونه‌گیری در دسترس بر روی ۶۰ بیمار مبتلا به پارگی لیگامان متقاطع قدامی ‌حاد انجام شد. به منظور ارزیابی آستانه حس تشخیص حرکت از دستگاه حرکت ممتد غیر‌فعال استفاده شد. این آزمون در سرعت ۰٫۵ درجه بر ثانیه انجام شد. متغیر پاسخ در این مطالعه آستانه حس تشخیص حرکت و متغیر‌‌های جراحی، بریس، چشم، اندام، BMI، سمت غالب، سمت ابتلا و سن نیز به‌عنوان متغیر‌‌های مستقل در نظر گرفته شدند. از نرم افزار R، نسخه ۲٫۱۳٫۰ جهت برازش مدل GEE ترکیبی استفاده گردید. یافته‌ها: جراحی بازسازی، در بهبود آستانه حس تشخیص حرکت بیماران موثر است. (۰/۰۰۱>P و ۲۵/۹۹۷–۷/۴۸۶ و %۹۵:CI و ۱۳/۹۴:OR)، ولی بریس عملکردی باعث بهبود آستانه حس تشخیص حرکت نمی‌شود (۰/۰۷۶=P و ۱/۰۳۳–۰٫۵۲۰و ٪۹۵:CI و ۰/۷۳:OR). همچنین بخت قرار گرفتن آستانه حس تشخیص حرکت بیماران با سمت غالب چپ، در وضعیت نرمال، در صورتی که سمت مبتلا راست باشد ۰/۰۱۹ برابر سمت چپ است (۰/۰۰۱>P و ۰٫۰۳۵–۰/۰۱۰ و ٪۹۵:CI و ۰/۰۱۹:OR) و عدم ارتباط معنی‌دار بین اندام بیماران و آستانه حس تشخیص حرکت نشان دهنده این است که اندام مبتلا، آستانه حس تشخیص حرکت اندام سالم را نیز تحت تأثیر قرار می‌دهد (۰/۷۲۸=P و ۱/۰۱۵–۰/۸۰۶ و ٪۹۵:CI و ۰/۹۷:OR). نتیجه‌گیری: از بین متغیر‌‌های موجود در مطالعه، متغیر‌‌های جراحی بازسازی، سمت ابتلا و اثر متقابل سمت ابتلا و سمت غالب رابطه معنی‌داری را نشان دادند.

Title: تآثیر برنامه‌های المپیک ویژه بر خودکارآمدی افراد سندرم داون

http://rehabilitationj.uswr.ac.ir/article-1-1093-fa.html

هدف: مطالعات تحقیقی نشان می‌دهند که شرکت در برنامه‌های اجتماعی نقش موثری در ارتقاء سلامت روانی و اجتماعی افراد کم‌توان‌ذهنی دارد. یکی از این برنامه‌ها، مسابقات ورزشی المپیک ویژه و برنامه‌های جانبی آن است. هدف از تحقیق حاضر بررسی تاثر برنامه‌های ورزشی و اجتماعی المپیک ویژه بر خودکارآمدی افراد سندرم داون می‌باشد. روش بررسی: ۴۰نفر ورزشکار (۱۶ دختر و ۲۴ پسر) سندرم داون که اعضای تیمهای ملی المپیک ویژه‌ ایران را تشکیل می‌دادند در این تحقیق شبه تجربی شرکت نمودند. افراد گروه کنترل نیز شامل ۲۲ پسر و ۱۵ دختر سندرم داون غیرورزشکار بودند که به‌طور تصادفی از انجمن‌های حمایت از افراد سندرم داون انتخاب شدند. ۹ ماه شرکت در برنامه‌های ورزشی-اجتماعی المپیک ویژه به عنوان پروتکل تمرینی گروه تجربی در نظر گرفته شد. ابزار تحقیق نیز شامل پرسشنامه خودکارآمدی ویلرولد بود. داده‌ها با آزمونهای آماری کولموگروف اسمیرنوف، تی‌زوجی و تی‌مستقل تحلیل شد. یافته‌ها: نتایج آزمون تی همبسته نشان داد که پس از نه ماه تمرین افزایش معنی‌داری در خودکارآمدی اجتماعی (۰/۰۰۱>P)، و خودکارآمدی عمومی (۰/۰۰۱>P ) در گروه تجربی حاصل شد، در حالیکه ‌این تغییر در گروه کنترل معنی‌دار نبود. نتیجه‌گیری: به نظر می‌رسد که شرکت در فعالیت‌های اجتماعی و ورزشی المپیک ویژه، می‌تواند حس خودکارآمدی را در این افراد افزایش داده و باعث گردد که در تعاملات اجتماعی‌شان وابستگی کمتری به والدین و دیگران داشته باشند.

Title: تأثیر آموزش اصول ارگونومی ‌بر اختلالات اسکلتی و عضلانی کاربران رایانه

http://rehabilitationj.uswr.ac.ir/article-1-1155-fa.html

هدف: این مطالعه با هدف بررسی تأثیر آموزش اصول ارگونومی بر اختلالات اسکلتی و عضلانی کاربران رایانه بهزیستی شهرستان رشت در سال ۹۰انجام شده است. روش بررسی: در این پژوهش نیمه آزمایشی ۲۸۳ کاربر رایانه زن که دارای اختلالات اسکلتی و عضلانی بودند به صورت تصادفی در دوگروه آزمایش (۲۳ نفر) و کنترل (۲۳ نفر (قرار گرفتند. سپس برای گروه آزمایش به مدت سه ماه آموزش‌های نظری و عملی و مداخلات ارگونومی ‌اجرا گردید.داده‌ها با استفاده از پرسش‌نامه نقشه بدن جمع‌آوری ونتایج باآزمون کولموگروف-اسمیرنوف و آزمون کوواریانس تحلیل شد. یافته‌ها: بیشترین شیوع میزان اختلالات اسکلتی-عضلانی در جامعه مورد نظر به ترتیب در زانوی راست (۲۷/۹۱ درصد)، گردن (۲۷/۲۰ درصد)، کمر و شانه راست (۲۶/۱۴ درصد)، زانوی چپ (۲۵/۷۹ درصد) و پشت (۲۳/۶۷ درصد) مشاهده شد. یافته‌های پژوهش بیانگر این است که پیش‌فرض همگنی شیب‌ها و همگنی واریانس‌ها تایید می‌گردد. آموزش‌های ارگونومیک منجر به کاهش معنادار اختلالات اسکلتی و عضلانی کاربران رایانه شد. نتیجه‌گیری:براساس یافته‌های پژوهش می‌توان نتیجه گرفت که آموزش و آگاه کردن کاربران رایانه از اصول ارگونومیک کار با رایانه و انجام مداخلات ارگونومیک جهت تصحیح نامناسب پوسچر،کاهش زمان کار، استفاده از تکیه‌گاه بازو و تکیه‌گاه پا و انجام تمرینات کششی و تنظیم ایستگاه کاری نقش موثری در کاهش اختلالات اسکلتی و عضلانی کاربران رایانه خواهد داشت.

Title: تأثیر برنامه منزل کاردرمانی بر فعالیت‌‌های روزمره زندگی در کودکان فلج‌مغزی ۵ تا ۱۲ ساله

http://rehabilitationj.uswr.ac.ir/article-1-1159-fa.html

هدف: فلج‌مغزی یکی از شایع‌ترین علل ناتوانی جسمی‌ در دوران کودکی است که منجر به مشکلات متعددی در فرد شده، سطح استقلال و مشارکت در انجام فعالیت‌‌های روزمره زندگی را کاهش می‌دهد. هدف این مطالعه بررسی تأثیر برنامه منزل کاردرمانی بر میزان عملکرد در فعالیت‌‌های روزمره زندگی در کودکان فلج‌مغزی ۵ تا ۱۲ ساله است. روش بررسی: در این کارآزمایی بالینی، ۴۸ کودک در دسترس به صورت تصادفی در دو گروه مداخله و شاهد تقسیم شدند. تمرینات متداول کاردرمانی طی ۴ ماه برای هر دو گروه انجام شد. گروه مداخله علاوه بر آن از آموزش برنامه منزل به صورت کارگاه و جزوات آموزشی به همراه پیگیری تلفنی استفاده کردند. به منظور ارزیابی فعالیت‌‌های روزمره از آزمون مقیاس سنجش فعالیت‌ها در کودکان (ASK) و به منظور تجزیه و تحلیل از آزمون‌‌های t مستقل، کای اسکوئر، تست دقیق فیشر و آنالیز کوواریانس استفاده شد. یافته‌ها: اختلاف معنادار در فعالیت‌‌های روزمره زندگی در دو گروه دیده شد (0/001=P). در تمامی‌ خرده‌مقیاس‌‌های فعالیت‌‌های روزمره زندگی-جز خرده آزمون‌ بازی کردن (P>0/05). نتیجه‌گیری: برنامه منزل کاردرمانی، باعث افزایش میزان فعالیت‌‌های روزمره زندگی در کودکان فلج‌مغزی می‌شود و می‌تواند در کنار برنامه‌‌های متداول کاردرمانی سودمند واقع شود.

Title: بررسی رابطه بین کیفیت زندگی و راهبردهای مقابله‌ای در بیماران اسکیزوفرنیک مراجعه‌کننده به درمانگاه‌های روانپزشکی بیمارستان‌های آموزشی تهران

http://rehabilitationj.uswr.ac.ir/article-1-1271-fa.html

هدف: عوامل زیادی هستند که کیفیت زندگی را تحت تأثیر قرار می‌دهند که از جمله می‌توان تنش‌زاها و روش‌های مقابله‌ای با آنها را نام برد. بیماران اسکیزوفرنیک از انواع تنش‌ها رنج برده و دارای مشکلاتی در راهبردهای مقابله‌ای خود در برخورد با تنش‌زاهای زندگی هستند که می‌تواند روی کیفیت زندگی آنها تأثیر بگذارد. لذا شناخت راهبردهای مقابله‌ای آنها دارای اهمیت می‌باشد. پژوهش حاضر با هدف بررسی راهبردهای مقابله‌ای مددجویان اسکیزوفرنیک و رابطه آن با کیفیت زندگی آنها انجام شد. روش بررسی: در این پژوهش توصیفی همبستگی ۹۰ بیمار اسکیزوفرنیک مراجعه‌کننده به درمانگاه‌های روانپزشکی بیمارستان‌های مراکز شهر تهران در یک مقطع زمانی ۹ ماهه با پرسش‌نامه‌های: راه‌های مقابله‌ای و نسخه فارسی پرسش‌نامه کیفیت زندگی سازمان بهداشت جهانی بررسی شدند. اطلاعات با آزمون‌های آنالیز واریانس یک‌طرفه، تی مستقل،کروسکال والیس، من وینتنی، کای دو و ضرایب همبستگی مورد تجزیه و تحلیل قرار گرفت. یافته‌ها: یافته‌ها نشان داد اکثریت واحدهای مورد پژوهش(٪۴۲/۱) از «راهبرد مقابله‌ای اجتنابی» استفاده می‌کردند و هم‌چنین اکثریت واحدهای مورد پژوهش (٪77/۸) دارای کیفیت زندگی متوسطی بودند. نتایج نشان داد که رابطه معنی‌داری بین میانگین نمرات کیفیت زندگی و نوع راهبردهای مقابله‌ای وجود دارد (۰/۰۰۵=P). به‌طوری‌که، واحدهای مورد پژوهشی که بیشتر از راهبردهای مقابله‌ای اجتنابی استفاده کرده بودند دارای میانگین امتیاز کلی کمتری در کیفیت زندگی بودند. نتیجه‌گیری: بین کیفیت زندگی و راهبردهای مقابله‌ای رابطه معنی‌داری وجود دارد که می‌توان با گنجاندن آموزش مهارت‌های مقابله‌ای در طرح درمان و توانبحشی بیماران اسکیزوفرنیک، توان آنها را در استفاده از راهبردهای مقابله‌ای موثر افزایش داده و کیفیت زندگی شان را ارتقاء داد.

Title: تأثیر برنامه نوتوانی قلب در بازسازی بطن چپ بیماران مرد و زن مبتلا به بیماری عروق کرونر

http://rehabilitationj.uswr.ac.ir/article-1-1258-fa.html

هدف: این پژوهش با استفاده از یافته‌های اکوکاردیوگرافی به بررسی تأثیر برنامه نوتوانی قلب بر بهبود فرآیند بازسازی قلب در بیماران مبتلا به بیماری عروق کرونری پرداخته و در نهایت نتایج بدست آمده در مردان و زنان را با یکدیگر مقایسه می‌کند. روش بررسی: از میان ۴۰ بیمار قلبی داوطلب، ۲۰ بیمار قلبی (۱۰ بیمار زن با میانگین سنی ۵٫۴۴±۵۴٫۵ و۱۰ بیمار مرد با میانگین سنی ۷٫۶۸±۵۳٫۲) که در مرحله دوم نوتوانی قلب قرار داشتند، در برنامه نوتوانی این پژوهش مداخله‌ای آینده نگر شرکت کردند. برنامه تمرین شامل تمرینات ورزشی استقامتی و مقاومتی به مدت ۶۰ دقیقه، ۳ جلسه در هفته و در مجموع به مدت ۲ ماه بود. قبل و بعد از برنامه نوتوانی، اکوکاردیوگرافی و آزمون ورزش در بیماران انجام شد. داده های حاصل با آزمون‌های آماری تی ‌زوجی و مستقل تحلیل شد. یافته‌ها: میزان کسر تخلیه‌ای در مردان از ۸±٪۵۲ به ۷٫۳۶±٪۶۰/۵ و در زنان از ۸±٪۴۹ به ۸/۲۲±٪۵۸/۵ افزایش یافت. میزان اندازه پایان دیاستولی بطن چپ در مردان از۶/۷۴ ±۵۰/۲۶ به ۵/۵۶±۴۵/۴ میلی‌متر و در زنان از ۴۷/۸۷±۱۱/۷۶ به ۸/۵۲±۴۴/۶۲ میلی‌متر و میزان اندازه پایان سیستولی بطن چپ در مردان از۷/۳۳±۳۲/۱۰ به ۸/۶±۸۶/۲۷ میلی‌متر و در زنان از ۹/۵۲±۳۳/۲۹ به ۶/۱±۲۸/۷۳ میلی‌متر کاهش یافت. میزان کسر کوتاه‌شدگی بطن چپ در مردان از ۵/۰۴±٪۲۷/۸ به ۵/۲۸±٪۳۸/۲۵ و در زنان از ۷/۶±٪۲۷/۳ به ۵/۵±٪۳۶/۱۵ افزایش داشت (P=۰/۰۰۱) لیکن آزمون آماری تی مستقل نشانگر تأثیر یکسان نوتوانی قلب در پیشگیری از بازسازی بطن چپ در هر دو جنس بود ).  نتیجه‌گیری: ۲۴ جلسه برنامه نوتوانی قلب تأثیر بارزی در پیشگیری از بازسازی بطن چپ در بیماران قلبی داشت که به نظر می‌رسد این تأثیر در دو گروه زن و مرد یکسان است.

Title: مقایسه خشونت تجربه شده در زنان دارای همسر معتاد به مواد افیونی و مواد محرک

http://rehabilitationj.uswr.ac.ir/article-1-1166-fa.html

هدف: مواد افیونی و مواد محرک دو گروه از مواد غیرقانونی هستند که از لحاظ ساختار شیمیایی، تأثیرات جسمانی و روانی و خطر بالقوه برای مصرف‌کننده، با یکدیگر تفاوت دارند. این پژوهش به دنبال آن است تا تفاوت تأثیرات سوءمصرف مواد افیونی و مواد محرک را در رفتار خشونت‌آمیز علیه همسران بررسی کند. &zwj&zwj&zwj روش بررسی: پژوهش کاربردی حاضر از نوع غیرتجربی و مقایسه‌ای است. نمونه پژوهش شامل ۱۰۰ نفر از همسران افراد معتاد به مواد افیونی و مواد محرک (۵۰ نفر مواد افیونی و ۵۰ نفر مواد محرک) بود که به منظور دریافت کمک‌های حرفه‌ای (از مهر تا دی ۹۰) به مراکز دولتی و غیردولتی و کمپ‌های استان تهران مراجعه کرده بودند. آزمودنی‌های هر دو گروه با روش نمونه‌گیری در دسترس انتخاب شدند. ابزار اندازه‌گیری استفاده شده برای ارزیابی میزان خشونت (روانشناختی، خشونت جنسی، مذاکره و گفتگو، خشونت فیزیکی و آسیب و صدمه) مقیاس تجدیدنظر شده تاکتیک‌های تعارضی (۲-CTS ) است. یافته‌ها با استفاده از تحلیل واریانس و کواریانس چند متغیره تحلیل شدند. یافته‌ها: بعد از کنترل سن به عنوان متغیر همپراش، مصرف‌کنندگان مواد محرک (مخصوصاً مت‌آمفتامین) خشونت بیشتری را نسبت به همسرانشان در مقایسه با مصرف‌کنندگان مواد افیونی نشان دادند.  (۰/۰۰۱>P) هم‌چنین دو گروه به جز مهارت‌های مذاکره، تفاوت معناداری در خشونت روانی، جنسی، حمله جسمانی و آسیب و صدمه داشتند. (P&le0.05) نتیجه‌گیری: بنظر می‌رسد خواص سایکوفارماکولوژی مواد محرک باعث می‌شود که افراد مصرف‌کننده مت‌آمفتامین خشونت بیشتری را نسبت به افراد مصرف‌کننده مواد افیونی داشته باشند.

Title: سندروم روبین اشتاین طیبی: ارزیابی تکاملی-گزارش موردی

http://rehabilitationj.uswr.ac.ir/article-1-1164-fa.html

سندروم روبین اشتاین طیبی یک ناهنجاری ژنتیکی نادری است که دارای ویژگی‌هایی از قبیل ناهنجاری‌های صورت، انگشت شصت پهن در دست و پا، قد و قامت کوچک و تأخیر تکاملی (حرکتی اعم از ریز و درشت، برقراری ارتباط، حل مساله، شخصی-اجتماعی) است. هدف این مقاله، گزارش پسری ۱۲ ماهه می‌باشد که به دلیل تأخیر تکامل به پزشک ارجاع شده و طی بررسیهای انجام شده سندرم روبین اشتاین طیبی برای وی تشخیص داده شد و همچنین گزارش نتایج ارزیابی تکاملی با تاکید بر بخش حرکتی با استفاده از دو آزمون، پرسشنامه سنین و مراحل (ASQ) و پی بادی۲ (PDMSII) بود.
